# Voriconazole and the Risk of Keratinocyte Carcinomas Among Lung Transplant Recipients in the United States

**DOI:** 10.1001/jamadermatol.2020.1141

**Published:** 2020-05-13

**Authors:** Monica E. D’Arcy, Ruth M. Pfeiffer, Donna R. Rivera, Gregory P. Hess, Elizabeth K. Cahoon, Sarah T. Arron, Isaac Brownell, Edward W. Cowen, Ajay K. Israni, Matthew A. Triplette, Elizabeth L. Yanik, Eric A. Engels

**Affiliations:** 1 Division of Cancer Epidemiology and Genetics, National Cancer Institute, Rockville, Maryland; 2 Division of Cancer Control and Population Sciences, National Cancer Institute, Rockville, Maryland; 3 The Wharton School, University of Pennsylvania, Philadelphia; 4 Department of Dermatology, University of California, San Francisco; 5 National Institute of Arthritis, Musculoskeletal and Skin Diseases, National Institutes of Health, Bethesda, Maryland; 6 Kidney Transplant Program, Hennepin Healthcare, University of Minnesota, Minneapolis; 7 Scientific Registry of Transplant Recipients, Minneapolis, Minnesota; 8 Clinical Research Division, Fred Hutchinson Cancer Research Center, University of Washington, Seattle; 9 Washington University in St Louis, St Louis, Missouri

## Abstract

**Importance:**

The antifungal medication voriconazole is used to prevent and treat aspergillosis, a major cause of mortality among recipients of lung transplants (hereinafter referred to as lung recipients). Small studies suggest that voriconazole increases risk of cutaneous squamous cell carcinoma (SCC).

**Objective:**

To examine associations of voriconazole and other antifungal medications with risk of keratinocyte carcinomas (SCC and cutaneous basal cell carcinoma [BCC]) in lung recipients.

**Design, Setting, and Participants:**

This population-based cohort study included non-Hispanic white patients (n = 9599) who underwent lung transplant in the United States from January 1, 2007, to December 31, 2016, identified through the national Scientific Registry of Transplant Recipients with data linkable to pharmacy claims. Data were analyzed from March 1, 2018, to February 13, 2019.

**Exposures:**

Antifungal medication use, including voriconazole, itraconazole, posaconazole, and other antifungals, was ascertained from pharmacy claims and treated as a time-varying exposure (assessed every 30 days). Cumulative antifungal exposure was calculated as the total number of exposed months.

**Main Outcomes and Measures:**

Primary outcomes were the first SCC or BCC reported to the transplant registry by transplant centers. Follow-up began at transplant and ended at SCC or BCC diagnosis, transplant failure or retransplant, death, loss to follow-up, or December 31, 2016. Cox proportional hazards regression models were used to estimate adjusted hazard ratios (AHRs) for each antifungal medication.

**Results:**

Among the 9793 lung transplants in 9599 recipients included in the analysis, median age at transplant was 59 (interquartile range [IQR], 48-65) years, 5824 (59.5%) were male, and 5721 (58.4%) reported ever smoking. During a median follow-up of 3.0 (IQR, 1.4-5.0) years after transplant, 1031 SCCs (incidence, 322 per 10 000 person-years) and 347 BCCs (incidence, 101 per 10 000 person-years) were reported. Compared with lung recipients with no observed voriconazole use, those with 1 to 3 months of voriconazole use experienced increased AHR for SCC of 1.09 (95% CI, 0.90-1.31); 4 to 7 months, 1.42 (95% CI, 1.16-1.73); 8 to 15 months, 2.04 (95% CI, 1.67-2.50); and more than 15 months, 3.05 (95% CI, 2.37-3.91). Ever itraconazole exposure was associated with increased SCC risk (AHR, 1.20; 95% CI, 1.00-1.45). For BCC, risk was not associated with voriconazole use but was increased with itraconazole use (AHR, 1.74; 95% CI, 1.27-2.37) or posaconazole use (AHR, 1.55; 95% CI, 1.00-2.41).

**Conclusions and Relevance:**

In this study, voriconazole use was associated with increased SCC risk among lung recipients, especially after prolonged exposure. Further research evaluating the risk-benefit ratio of shorter courses or alternative medications in transplant recipients at high risk for SCC should be considered.

## Key Points

**Question:**

What is the association between voriconazole, an antifungal used to treat aspergillosis infections, and the risk of keratinocyte carcinomas among recipients of lung transplants?

**Findings:**

In a population-based cohort study of 9599 non-Hispanic white recipients of 9793 lung transplants in the United States (2007-2016) with linkage to pharmacy claims, increasing cumulative voriconazole exposure was associated with an increased risk of cutaneous squamous cell carcinoma.

**Meaning:**

These findings suggest that physicians caring for lung transplant recipients at high risk for aggressive keratinocyte carcinomas should limit voriconazole exposure when possible and encourage skin protection behaviors and more frequent cancer screenings.

## Teaser

This population-based cohort study examines associations of voriconazole and other antifungal medications with risk of keratinocyte carcinomas in recipients of lung transplants.

## Introduction

Solid organ transplant provides potentially curative treatment for patients with end-stage organ disease. The number of transplants has grown over time, with 34 770 solid organ transplants occurring in the United States in 2017, of which 2478 were lung transplants.^1,2^ Solid organ transplant recipients have elevated risk for infections as well as many types of cancer, particularly virus-related cancers, which largely results from the immunosuppression caused by medications used to prevent graft rejection.^3,4^

For unclear reasons, transplant recipients have a strongly elevated risk for keratinocyte carcinomas (KCs), which comprise cutaneous squamous cell carcinoma (SCC) and basal cell carcinoma (BCC).^5^ Notably, cutaneous SCC is the most common cancer among transplant recipients and causes substantial morbidity, with rates especially elevated among recipients of lung transplants (hereinafter referred to as lung recipients).^6^ Keratinocyte carcinoma risk factors among transplant recipients include white race (particularly for individuals with Fitzpatrick skin types I-III^7^), residence in regions with high ambient UV radiation (UVR), older age, and history of skin cancer.^5,6,8^ Although immunosuppression likely plays a role, SCC is not known to be caused by a virus, and risk is increased to a much smaller degree in immunosuppressed individuals with HIV infection.^9^

Severe fungal infections are a major cause of mortality among lung recipients.^10^ Voriconazole is a broad-spectrum, oral triazole antifungal medication that was first approved in the United States in May 2002. It is commonly given to lung and allogeneic hematopoietic stem cell recipients to prevent and treat invasive aspergillosis. Voriconazole prophylaxis is often administered immediately after transplant for a period ranging from several months to more than 1 year, and voriconazole may also be used preemptively after detection of aspergillus colonization.^11,12,13^ Voriconazole is currently the first-line agent for prevention and treatment of aspergillus infections in lung recipients based on high-quality evidence.^13^

Voriconazole is associated with adverse events, including skin phototoxicity reactions,^14,15,16,17,18^ and case reports have described aggressive cutaneous SCCs occurring in solid organ and bone marrow transplant recipients during voriconazole treatment.^14,15,16^ These reports led to a few modest-sized studies of solid organ transplant recipients (range, 68-543 recipients, which included a range of 17-86 SCC cases),^14,19,20,21,22,23,24^ most of which demonstrated an association between voriconazole and increased SCC risk. In the largest and most recent study, Hamandi et al^25^ evaluated 900 lung recipients at 14 centers who underwent transplant from 2005 to 2008. Based on a total of 55 SCC cases, the investigators documented an association between any current or previous (ie, ever) voriconazole exposure and cutaneous SCC (adjusted hazard ratio [AHR], 2.39; 95% CI, 1.31-4.37). A recent meta-analysis^26^ that included 1521 lung recipients and 185 SCCs also indicated an increased risk of SCC associated with ever use of voriconazole (relative risk, 1.65; 95% CI, 1.02-2.68) and duration of use (relative risk per 180 days of use, 1.74; 95% CI, 0.95-3.18).

No large population-based study of voriconazole and cutaneous SCC among transplant recipients has been performed, and with the exception of the study by Hamandi et al,^25^ previous studies^14,19,20,21,22,23,24^ were small and performed at a single transplant center. Generalizing results of studies performed at a single site or a small number of sites is challenging, because transplant centers use diverse posttransplant treatment protocols. Moreover, only 1 study^27^ examined the association between voriconazole and cutaneous BCC in lung recipients. That study reported no association. Finally, previous studies were underpowered to examine the association between newer antifungal agents, including posaconazole,^20^ and KC risk.

The goal of the present study was to examine the association between voriconazole use and both SCC and BCC in a large population-based cohort of lung recipients. An ancillary aim was to examine the association between clinical alternatives to voriconazole, such as posaconazole, and KC risk.

## Methods

This cohort study was approved by the human subjects committee at the National Cancer Institute, which waived the need for informed consent for use of registry data collected for surveillance and research purposes. We used data from the Scientific Registry of Transplant Recipients (SRTR), which captures all US solid organ transplants, linked with outpatient pharmacy claims for 2007 to 2016 from Symphony Health, a nationwide aggregator of health insurance claims. The SRTR provided information on incident SCC and BCC collected from transplant centers as part of required reporting of posttransplant malignant neoplasms. Because this ascertainment has low sensitivity (estimated, 14%-41%),^6,28^ we missed a large proportion of KC cases. However, SRTR KC reporting has high positive predictive value (71%-88%) and specificity (99% for SCC). We obtained Symphony Health claims for immunosuppressant medications (include tacrolimus, cyclosporine, mycophenolate mofetil, azathioprine, sirolimus, everolimus, and corticosteroids) and antifungals (voriconazole, itraconazole, posaconazole, and other antifungals). This study followed the Strengthening the Reporting of Observational Studies in Epidemiology (STROBE) reporting guideline.

Our study included non-Hispanic white individuals who underwent lung transplantation from January 1, 2007, to December 31, 2016, because our preliminary analyses indicated that claims for immunosuppressant medications were available for approximately 80% of recipients from 2007 onward. Other racial/ethnic groups experienced fewer than 5 outcomes and were thus excluded from the study. For included recipients, follow-up began at transplant and ended at the first cancer diagnosis (SCC or BCC, depending on the analysis), transplant failure or retransplant, death, loss to follow-up, or end of study (December 31, 2016). For each specific analysis, the other outcome (SCC or BCC) was ignored rather than censored. If a person had multiple successive transplants, each transplant was treated as a separate period of follow-up.

Follow-up was partitioned into 30-day intervals beginning at transplant. We assigned each medication claim a start date equal to the dispensed date and an end date equal to the start date plus the days’ supply plus a 15-day grace period to account for small gaps in use or pill splitting. Exposure status for each medication was ascertained at the beginning of each 30-day interval. Because all recipients must use maintenance immunosuppressant medications to prevent rejection, we assumed that lack of immunosuppressant claims implied incomplete pharmacy claims information for an interval. We then excluded transplant recipients who had documented immunosuppressant coverage for less than 25% of their 30-day follow-up intervals.

Because many included recipients lacked immunosuppressant claims for some 30-day intervals, our classification of antifungal medication exposure included an unknown category. Specifically, a person was considered exposed to an antifungal medication if a claim indicated use of that medication, and unexposed if no claim for the antifungal was observed but a claim for at least 1 immunosuppressant medication was observed. We then assigned unknown antifungal exposure status for an interval if there was no claim for the antifungal medication and no claim for an immunosuppressant.

On the basis of the assignments for each 30-day interval, we created time-dependent variables that captured use of each antifungal medication at the start of each interval. Ever-exposed status was assigned if the current interval or any prior interval indicated exposure to that medication. If no current or prior interval had an exposed status and more than 75% of all current or prior intervals had unexposed status, we assigned never-exposed status. This approach was necessary because a large proportion of the population had unknown exposure status during their first interval (likely resulting from initial inpatient receipt of medications, which is not captured by the Symphony Health pharmacy claims). Thus, a person could transition from unknown to unexposed status once a preponderance of claims indicated lack of exposure. All other intervals were classified as having unknown exposure status. For descriptive purposes, we present estimates of antifungal medication use according to time since transplant and calendar year of transplant. Specifically, the proportion of the cohort exposed to each antifungal medication in these categories was calculated as the proportion of exposed person-time divided by the known person-time (ie, exposed or unexposed).

Data were analyzed from March 1, 2018, to February 13, 2019. From the SRTR, we obtained data on recipient demographic and transplant characteristics. Mean annual ambient exposure to UVR was derived by mapping recipient residence as previously described.^29^ Univariate associations were examined between these variables and SCC or BCC in separate Cox proportional hazards regression models. We used time since transplant as the timescale and stratified the baseline hazard by calendar year of transplant (in 2-year increments). Variables that were statistically significantly associated with the outcome were then included in a multivariable model, and those that remained associated with the outcome after mutual adjustment were retained for a final base model (eMethods in the Supplement). We included ambient UVR exposure in the final models regardless of statistical significance because of voriconazole’s documented photosensitizing effects.^14^

We then used Cox proportional hazards regression models to assess associations between antifungal medications (treated as time-dependent variables) and the risk of SCC or BCC. We evaluated current or prior antifungal exposure using variables that captured ever, never, or unknown and cumulative exposure based on the total number of exposed intervals (unknown intervals did not contribute to this calculation). We also considered different intervals to lag the exposure (0, 180, and 360 days) to assess delayed effects of the medications. When cumulative exposure of an antifungal medication was associated with the outcome, we report results for both the continuous measure (per 30-day interval) and based on percentile categories of use (approximately <33rd, 33rd-66th, 66th-90th, and >90th percentiles).

## Results

A total of 18 144 first or subsequent lung transplants were performed in the United States from 2007 to 2016, of which 16 180 were performed in non-Hispanic white individuals. After excluding 6371 transplants with immunosuppressant coverage for less than 25% of 30-day intervals and 16 transplants lacking smoking information or mappable UVR exposure, we included 9793 lung transplants among 9599 unique individuals in the present study (Table 1).

**Table 1.** Baseline Characteristics, Follow-up, and Antifungal Medication Exposure Status of Lung Transplants Included in the Study

Characteristic	Lung transplants (n = 9793)^a^
Age at transplant, median (IQR), y	59 (48-65)
Sex	
Female	3969 (40.5)
Male	5824 (59.5)
Smoking status	
Never	4072 (41.6)
Current or former	5721 (58.4)
Calendar year of transplant	
2007-2010	3346 (34.2)
2011-2013	3147 (32.1)
2014-2016	3300 (33.7)
Indication for transplant	
Chronic obstructive pulmonary disease	2369 (24.2)
Idiopathic pulmonary fibrosis	3555 (36.3)
Other^b^	3869 (39.5)
Transplant No.	
First	9325 (95.2)
Second or greater	468 (4.8)
Procedure type	
Double lung	6791 (69.3)
Single lung	3002 (30.7)
Received induction therapy	
No	4198 (42.9)
Yes	5595 (57.1)
Daily mean annual ambient UVR, mW/m^2^	
<26.00	2465 (25.2)
26.00-31.99	2389 (24.4)
32.00-46.99	2654 (27.1)
≥47.00	2285 (23.3)
Duration of follow-up, median (IQR), y	3.0 (1.4-5.0)
30-d Intervals with claim for maintenance immunosuppressant medication, median (IQR), %^c^	75.0 (51.4-89.9)
Antifungal use	
Voriconazole	4002 (40.9)
Itraconazole	2573 (26.3)
Posaconazole	1232 (12.6)
Other^d^	2124 (21.7)

Abbreviations: IQR, interquartile range; UVR, UV radiation.

^a^ Unless otherwise indicated, data are expressed as number (percentage) of transplants.

^b^ Includes cystic fibrosis, other obstructive lung diseases, inflammatory and fibrotic lung, airway diseases, pulmonary hypertension and pulmonary vascular diseases, and other or unspecified conditions.

^c^ Includes tacrolimus, cyclosporine, mycophenolate mofetil, azathioprine, sirolimus, everolimus, and corticosteroids.

^d^ Includes fluconazole, amphotericin B, caspofungin acetate, micafungin sodium, anidulafungin, and isavuconazonium sulfate.

Among included transplants, the median recipient age at transplant was 59 (interquartile range [IQR], 48-65) years, 5824 (59.5%) were male, 3969 (40.5%) were female, and 5721 (58.4%) reported ever smoking. Idiopathic pulmonary fibrosis (3555 [36.3%]) and chronic obstructive pulmonary disease (2369 [24.2%]) were the most common reasons for transplant. Most of the procedures (9325 [95.2%]) were first transplants, and 6791 (69.3%) were double lung transplants. Induction therapy was documented for 5595 transplants (57.1%). Median follow-up was 3.0 (IQR, 1.4-5.0) years after transplant, and the median proportion of 30-day intervals with maintenance immunosuppressant claims was 75.0% (IQR, 51.4%-89.9%). Demographic characteristics of the excluded transplants were similar, except follow-up was shorter (median, 2.0 [IQR, 0.7-4.0] years) (eTable 1 in the Supplement).

**Figure 1.**
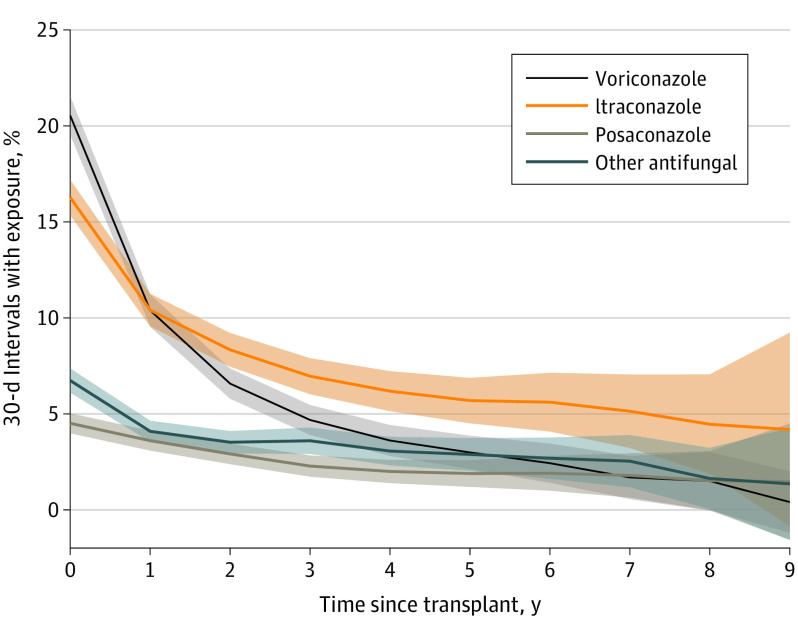
Antifungal Medication Exposure as a Function of Time Since Transplant Exposure is measured as a claim for the specified antifungal medication observed. The percentages are calculated using only intervals with known exposure status. The other antifungal medication category includes the following medications: fluconazole, amphotericin B, caspofungin acetate, micafungin sodium, anidulafungin, and isavuconazonium sulfate. Shaded areas indicate 95% CI.

**Figure 2.**
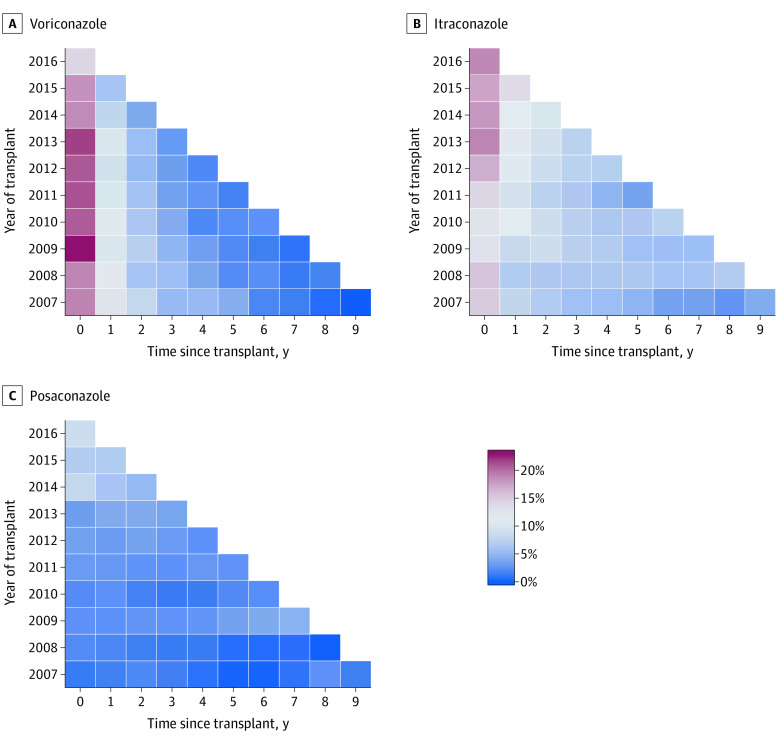
Antifungal Medication Exposure as a Function of Transplant Calendar Year and the Time Since Transplant Medication use for each lung transplant was assessed every 30 days (interval), and the colors within the boxes represent the proportion of intervals in which an antifungal medication was observed for the specific time since transplant–calendar year combination. The darkest red boxes correspond to more than 20% and the darkest blue boxes to less than 5% of intervals. The percentages are calculated using only intervals with known exposure status.

Overall, 4002 lung transplants (40.9%) had at least 1 claim for voriconazole, followed by itraconazole at 2573 (26.3%) and posaconazole at 1232 (12.6%) (Table 1). As shown in Figure 1 and Figure 2, antifungal medication use was greatest in the first year after transplant, when lung recipients are at highest risk of fungal infections. Voriconazole and itraconazole were the most commonly used antifungals in the first year (20.5% and 16.3% of person-time). Voriconazole use dropped rapidly from 20.5% in the first year after transplant to 10.4% in the second year, and itraconazole was more commonly used than voriconazole 2 or more years after transplant (Figure 1). Voriconazole use decreased slightly over calendar time (for example, as shown in Figure 2, use in the first year after transplant decreased from 19.1% in 2007 to 15.9% in 2016), whereas no clear patterns for itraconazole use by calendar time emerged. Although posaconazole use was less common than itraconazole and voriconazole use, it exhibited a more gradual decline with greater time since transplant (4.5% in the first year to 1.5% in the tenth year) compared with voriconazole (20.5% in the first year to 0.4% in the tenth year) and itraconazole (16.3% in the first year to 2.5% in the tenth year) (Figure 1). Posaconazole use also increased over calendar time; for example, its use increased from 0.9% in the first year since transplant in 2007 to 8.8% in the first year since transplant in 2016 (Figure 2).

A total of 1031 cutaneous SCCs were observed during follow-up (incidence, 322 per 10 000 person-years). Based on our model-fitting procedure (eResults in the Supplement), our final model for SCC included cumulative voriconazole exposure assessed without a lag (Table 2). Individuals with voriconazole exposure experienced increasing SCC risk with AHRs of 1.09 (95% CI, 0.90-1.31) with 1 to 3 months, 1.42 (95% CI, 1.16-1.73) with 4 to 7 months, 2.04 (95% CI, 1.67-2.50) with 8 to 15 months, and 3.05 (95% CI, 2.37-3.91) with more than 15 months of exposure, compared with those with no voriconazole exposure. Similarly, for each 30-day voriconazole exposure, SCC risk increased by 5% (AHR, 1.05; 95% CI, 1.04-1.06). As shown in Table 2, other factors associated with SCC included male sex (AHR, 2.01; 95% CI, 1.73-2.32), increasing age category (AHR, 1.54; 95% CI, 1.44-1.65), indication for transplant (eg, AHR for idiopathic pulmonary fibrosis compared with chronic obstructive pulmonary disease, 1.44; 95% CI, 1.23-1.69), second or subsequent transplant (AHR, 2.49; 95% CI, 1.92-3.23), and smoking history (AHR, 1.28; 95% CI, 1.10-1.49). Although itraconazole was not significantly associated with SCC when initially assessed (eTable 2 in the Supplement), there was a modest and borderline significant association with ever use in the final model (AHR, 1.20; 95% CI, 1.00-1.45). Exposure to posaconazole, other antifungals, and UVR were not associated with SCC in the multivariate model (Table 2).

**Table 2.** Risk Factors for Cutaneous SCC Among 9793 Lung Transplants

Characteristic	AHR (95% CI)^a^
Sex	
Female	1 [Reference]
Male	2.01 (1.73-2.32)
Age at transplant per category^b^	1.54 (1.44-1.65)
Indication for transplant	
Chronic obstructive pulmonary disease	1 [Reference]
Idiopathic pulmonary fibrosis	1.44 (1.23-1.69)
Other^c^	1.28 (1.05-1.55)
Smoking history	
No	1 [Reference]
Yes	1.28 (1.10-1.49)
Daily mean annual ambient UVR, mW/m^2^	
<26.00	1 [Reference]
26.00-31.99	0.94 (0.79-1.13)
32.00-46.99	0.94 (0.79-1.12)
≥47.00	1.11 (0.94-1.32)
Transplant No.	
First	1 [Reference]
Second or greater	2.49 (1.92-3.23)
Duration of voriconazole use^d^	
No use	1 [Reference]
1-3 mo	1.09 (0.90-1.31)
4-7 mo	1.42 (1.16-1.73)
8-15 mo	2.04 (1.67-2.50)
>15 mo	3.05 (2.37-3.91)
Ever itraconazole use	
No	1 [Reference]
Yes	1.20 (1.00-1.45)
Unknown	0.94 (0.73-1.21)
Ever posaconazole use	
No	1 [Reference]
Yes	1.08 (0.83-1.39)
Unknown	1.02 (0.75-1.40)
Ever other antifungal use^e^	
No	1 [Reference]
Yes	1.10 (0.89-1.36)
Unknown	1.32 (0.98-1.76)

Abbreviations: AHR, adjusted hazard ratio; SCC, squamous cell carcinoma; UVR, UV radiation.

^a^ Cox proportional hazards regression models are adjusted for all variables in the table. The baseline hazard was stratified by calendar year of transplantation (2007-2008, 2009-2010, 2011-2012, 2013-2014, and 2015-2016).

^b^ Categorized as an ordinal variable corresponding to the following ranges: less than 30, 30 to 39, 40 to 49, 50 to 59, 60 to 69, and older than 69 years.

^c^ Includes cystic fibrosis, other obstructive lung diseases, inflammatory and fibrotic lung, airway diseases, pulmonary hypertension and pulmonary vascular diseases, and other or unspecified conditions.

^d^ Assessed in 30-day increments; duration of use categories correspond to approximately less than the 33rd, 33rd to 66th, 66th to 90th, and greater than 90th percentiles.

^e^ Includes fluconazole, amphotericin B, caspofungin acetate, micafungin sodium, anidulafungin, and isavuconazonium sulfate.

There were 347 BCCs observed during follow-up (incidence, 101 per 10 000 person-years). Our model-fitting procedure identified ever use of itraconazole and ever use of posaconazole as risk factors for BCC, but there was no association with cumulative exposure (eTable 3 in the Supplement); the model with no lag fit the data well. In the final model (Table 3), ever exposures to itraconazole (AHR, 1.74; 95% CI, 1.27-2.37) and posaconazole (AHR, 1.55; 95% CI, 1.00-2.41) were associated with increased BCC risk. Other variables associated with BCC in this final model included male sex (AHR, 1.62; 95% CI, 1.28-2.06), increasing age category (AHR, 1.37; 95% CI, 1.23-1.52), and a history of smoking (AHR, 1.33; 95% CI, 1.03-1.72).

**Table 3.** Risk Factors for Cutaneous BCC Among 9793 Lung Transplants

Characteristic	AHR (95% CI)^a^
Sex	
Female	1 [Reference]
Male	1.62 (1.28-2.06)
Age at transplant per category^b^	1.37 (1.23-1.52)
Smoking history	
No	1 [Reference]
Yes	1.33 (1.03-1.72)
Daily mean annual ambient UVR, mW/m^2^	
<26.00	1 [Reference]
26.00-31.99	0.79 (0.59-1.06)
32.00-46.99	0.68 (0.50-0.92)
≥47.00	1.02 (0.77-1.36)
Ever voriconazole use	
No	1 [Reference]
Yes	0.88 (0.64-1.21)
Unknown	0.92 (0.59-1.44)
Ever itraconazole use	
No	1 [Reference]
Yes	1.74 (1.27-2.37)
Unknown	1.18 (0.77-1.81)
Ever posaconazole use	
No	1 [Reference]
Yes	1.55 (1.00-2.41)
Unknown	1.52 (0.87-2.66)
Ever other antifungal use^c^	
No	1 [Reference]
Yes	0.90 (0.60-1.35)
Unknown	0.75 (0.45-1.24)

Abbreviations: AHR, adjusted hazard ratio; BCC, basal cell carcinoma; UVR, UV radiation.

^a^ Cox proportional hazards regression models are adjusted for all variables in the table. The baseline hazard was stratified by calendar year of transplantation (2007-2008, 2009-2010, 2011-2012, 2013-2014, and 2015-2016).

^b^ Categorized as an ordinal variable corresponding to the following age ranges: less than 30, 30 to 39, 40 to 49, 50 to 59, 60 to 69, and greater than 69 years.

^c^ Includes fluconazole, amphotericin B, caspofungin acetate, micafungin sodium, anidulafungin, and isavuconazonium sulfate.

## Discussion

In this population-based study of lung recipients, voriconazole exposure was strongly associated with SCC risk, such that individuals with more than 15 months of exposure experienced 3-fold increased risk of SCC compared with unexposed individuals. Although itraconazole was also associated with SCC, the association was modest in magnitude and of borderline significance, and no association with duration of use was found. In addition, both itraconazole and posaconazole were associated with BCC risk, but neither was associated with BCC in a cumulative fashion.

Our results showing an association between cumulative voriconazole exposure and SCC risk are similar to those reported by Hamandi et al^25^ for lung recipients and in a recent meta-analysis of studies of lung and hematopoietic stem cell transplant recipients.^26^ Our study of 9793 lung transplants is much larger than prior studies and included 1031 SCC cases compared with 55 in Hamandi et al^25^ and 185 in the meta-analysis.^26^ This large size allowed us to model the association with increasing duration of voriconazole and adequately examine associations with clinical alternatives to voriconazole. Similar to previous reports,^21,22,25,27,30^ we found that increasing age and male sex were risk factors for SCC and BCC. Smoking history, which was not assessed in prior studies, was also positively associated with SCC and BCC in the present study. Exposure to UVR was included as a potential confounder, although it was not significantly associated with skin cancer in the multivariate models.

Although voriconazole can cause severe phototoxic reactions,^15,16,17,18^ carcinogenic mechanisms are poorly understood. Voriconazole exposure modifies keratinocyte gene expression, specifically affecting genes related to the cell cycle, including the *FOXM1* pathway—which is associated with SCC—and terminal differentiation pathways.^30^ Voriconazole’s primary metabolite (voriconazole n-oxide) and UV-A radiation can act together to damage DNA and inhibit apoptosis.^31^ Moreover, voriconazole can induce upregulation of cyclooxygenase 2, which is critical to SCC progression.^31^

In contrast to our findings for SCC, we found no evidence of an association between voriconazole and BCC. Our BCC results are also comparable to those of the recent meta-analysis,^26^ but the meta-analysis included only 2 studies with a total of 41 BCC cases. Both itraconazole and posaconazole were associated with increased BCC risk, although we did not find a cumulative effect. The positive associations are puzzling because no other evidence links either medication to BCC risk, and limited evidence suggests that both may actually limit BCC progression.^32,33,34^ We speculate that our BCC results may be due to confounding by physician prescribing preferences. After publication of reports documenting a possible association between voriconazole and aggressive skin cancer first appearing in 2007^15^ and 2010,^16^ physicians may have channeled high-risk patients (eg, those with fair skin or a history of skin cancer) away from voriconazole and toward alternative medications. This pattern of prescribing, if present, may also explain the small increased risk for SCC associated with itraconazole. Moreover, it might also have led us to underestimate the voriconazole-SCC association if patients less susceptible to SCC were preferentially prescribed voriconazole.

Antifungal medication use was highest in the first few years after transplant, corresponding to when lung recipients are at highest risk of aspergillosis and other fungal infections. Interestingly, voriconazole was the most commonly used antifungal immediately after transplant, but use declined precipitously compared with other antifungals after the first year after transplant, which may reflect physicians’ perception, based on earlier studies, that longer voriconazole use increases SCC risk.^22,25^ Voriconazole use decreased during the study period, and there appeared to be an increase over time in use of posaconazole, a clinical alternative to voriconazole that received US Food and Drug Administration approval in late 2006.

### Strengths and Limitations

Our study has several notable strengths. It is population based and the largest study to date, including 61% of all lung transplants among non-Hispanic white recipients in the United States during 2007 to 2016. The study’s large size enabled us to statistically adjust for all measured risk factors and assess alternative antifungal medications to voriconazole. By linking pharmacy claims, we had precise medication exposure information. Incorporation of a grace period allowed for late medication pickup and pill splitting, and our requirement for immunosuppressant coverage enhanced confidence in the classification of antifungal use. In addition, smoking history, an SCC risk factor^35^ not assessed in previous studies, was largely complete in our data.

Nonetheless, some limitations should be considered. We lacked data on skin cancers before transplant. As previously mentioned, physicians may have chosen antifungal medications based on this history, which could have biased associations, most likely in a conservative direction with respect to voriconazole. We assessed mean ambient UVR exposure based on recipients’ residence at the time of transplant.^29^ However, this measure does not capture previous residences, time spent outdoors, or sun protection behaviors, which may explain why we did not see strong associations between this measure and risk of KC.

Our linkage to pharmacy information was incomplete for some individuals and was commonly missing immediately after lung transplant, when they were likely in the hospital or using medications that they brought home with them. We attempted to mitigate this limitation by creating a category for unknown antifungal exposure status when immunosuppressant claims were not observed for a given interval. We also assigned individuals who began with unknown antifungal status to never-exposed status after they accumulated sufficient unexposed intervals (>75% of their previous intervals), which increased the likelihood that these individuals were truly unexposed.

Finally, SCC and BCC were ascertained from SRTR based on transplant center reports. Although this ascertainment has low sensitivity, the positive predictive value was high, which supports the validity of the captured cases. As a result of this misclassification, the AHRs may be slightly attenuated and our associations somewhat conservative. In addition, we lacked information on identified cancer features and thus cannot determine whether voriconazole is associated with deadly or low-risk SCC.

## Conclusions

In this study, voriconazole was associated with increased SCC risk among lung recipients, especially after prolonged exposure. Voriconazole is a highly effective treatment for aspergillosis—a major source of mortality among lung recipients—and is thus the recommended first-line treatment against suspected or confirmed infections.^13^ Although the efficacy of antifungal medications is the key consideration for the treatment of established infections, the balance of benefits and risks likely differs for prophylaxis, and broadly accepted guidelines are lacking.^36^ Given the high morbidity associated with SCC in the transplant setting, it is reasonable that physicians should consider the risk-benefit ratio of shorter durations of voriconazole prophylaxis or using alternatives to voriconazole in patients with high KC risk, encourage screening, and advise them about sun protective measures. Future research should focus on identifying the minimal time necessary for effective prophylaxis in lung recipients and quantifying the risks and benefits of prophylaxis with various medications in specific populations, including further assessment of posaconazole and itraconazole as alternatives with potentially lower SCC risk.

## Notes

Supplement. eMethods. Model Fitting eResults. Outcome eTable 1. Baseline Characteristics and Follow-up of Excluded 6387 Transplants eTable 2. Association Between Demographic or Clinical Factors and Cutaneous SCC Among Individuals With at Least 360 Days of Follow-up, With Medication Exposure Lagged 360 Days eTable 3. Association Between Demographic or Clinical Factors and Cutaneous BCC Among Individuals With at Least 360 Days of Follow-up, With Medication Exposure Lagged 360 Days
