# Supplementary material for: Voriconazole and the Risk of Keratinocyte Carcinomas Among Lung Transplant Recipients in the United States
Source: JAMA Dermatol. 2020 May 13;156(7):1–9. doi: 10.1001/jamadermatol.2020.1141 (PMC7221851; doi:10.1001/jamadermatol.2020.1141)
Supplement: Supplement. — eMethods. Model Fitting eResults. Outcome eTable 1. Baseline Characteristics and Follow-up of Excluded 6387 Transplants eTable 2. Association Between Demographic or Clinical Factors and Cutaneous SCC Among Individuals With at Least 360 Days of Follow-up, With Medication Exposure Lagged 360 Days eTable 3. Association Between Demographic or Clinical Factors and Cutaneous BCC Among Individuals With at Least 360 Days of Follow-up, With Medication Exposure Lagged 360 Days [file jamadermatol-156-772-s001.pdf]

Supplementary Online Content

D'Arcy ME, Pfeiffer RM, Rivera DR, et al. Voriconazole and the risk of keratinocyte carcinomas among lung transplant recipients in the United States. *JAMA Dermatol*. Published online May 13, 2020. doi:10.1001/jamadermatol.2020.1141

eMethods. Model Fitting
eResults. Outcome
eTable 1. Baseline Characteristics and Follow-up of Excluded 6387 Transplants
eTable 2. Association Between Demographic or Clinical Factors and Cutaneous SCC Among Individuals With at Least 360 Days of Follow-up, With Medication Exposure Lagged 360 Days
eTable 3. Association Between Demographic or Clinical Factors and Cutaneous BCC Among Individuals With at Least 360 Days of Follow-up, With Medication Exposure Lagged 360 Days

This supplementary material has been provided by the authors to give readers additional information about their work.

© 2020 D'Arcy ME et al. *JAMA Dermatology.*

eMethods. Model Fitting

We first assessed each antifungal medication in multivariate models using the variable indicating ever/never/unknown current or prior exposure (see Methods in paper). For those antifungal medications associated with the outcome, we added to the model the corresponding cumulative exposure variable and used a likelihood ratio test (LRT) to evaluate whether the cumulative exposure term improved the model. If so, then a final LRT was used to examine if the ever/never/unknown exposure variable could be removed to create a simpler model. These exposure variables were assessed initially in our cohort starting follow-up at 360 days post-transplant, to evaluate whether the medications were associated with KC risk incorporating a lag of 360 days. Once we determined the appropriate exposure variable (ever/never/unknown and/or cumulative exposure), we then identified the most appropriate lag using the Akaike information criterion (AIC). If a shorter lag was selected (180 days or no lag), then we refitted the final model to the corresponding cohort with follow-up starting at 180 days or 0 days after transplantation.

eResults. Outcome

SCC model

The fully adjusted model, which was determined using a 360-day lag for antifungal medication exposures, included age at transplantation, sex, smoking, indication for transplantation, transplant number, and UVR exposure (Supplemental Table 2). Among antifungal medications, only voriconazole was associated with SCC risk (adjusted hazard ratio [aHR] 1.57, 95%CI 1.29-1.92, for ever-exposed compared with never-exposed status). For voriconazole, addition of the cumulative exposure variable improved the model ($p=4.5*10^{-12}$), and the ever/never/unknown variable was then removed because it was no longer significant (p=0.29). The model that included cumulative voriconazole exposure with 0 days lag fit the data better than the model with 360-day lag (AIC 11686 vs. 11698). Therefore, the final model for SCC was refitted to the full cohort with follow-up starting at transplantation (Table 2).

BCC model

The fully adjusted model incorporating a 360-day lag for antifungal medications included age, sex, smoking, and UVR exposure (Supplemental Table 3). Among antifungal medications, only itraconazole and posaconazole were associated with BCC; however, neither cumulative itraconazole nor cumulative

© 2020 D'Arcy ME et al. *JAMA Dermatology.*

posaconazole use was associated with BCC (p=0.17 and 0.39, respectively). Because the model with 0 days lag fit the data as well as for 360 days lag (AIC 3926 vs. 3924), we refit the model to the full cohort with follow-up starting at transplantation.

© 2020 D'Arcy ME et al. *JAMA Dermatology.*

eTable 1. Baseline Characteristics and Follow-up of Excluded 6387 Transplants

**Characteristic**		
Age at transplantation in years, median (IQR)	60	(50,65)
Sex, N (%)		
Female	2,444	38.3
Male	3,943	61.7
Smoking Status, N (%)		
No	2,602	40.7
Yes	3,775	59.1
Calendar year of transplantation, N (%)		
2007-2010	2,341	36.7
2011-2013	1,775	27.8
2014-2016	2,271	35.6
Indication for transplantation, N (%)		
Chronic obstructive pulmonary disease	1,577	24.7
Idiopathic pulmonary fibrosis	2,338	36.6
All other	2,472	38.7
Transplant number, N (%)		
First	6,080	95.2
Second or higher	307	4.8
Procedure type, N (%)		
Double lung	4,234	66.3
Single lung	2,153	33.7
Reveived induction therapy, N (%)		
No	3,175	49.7
Yes	3,212	50.3
Duration of follow-up in years, median (IQR)	2.0	(0.7,4.0)
Percentage of 30-day intervals with claim for maintenance immunouppresant medication [a], median (IQR)	0.0	(0,10.9)

Abbreviations: IQR, interquartile range
Occurring in non-hispanic white persons excluded from the study because they had fewer than 25% of follow-up intervals with documented immunosuppressant coverage, or were missing smoking or UVR information

a. Maintenance immunosuppressant medications include tacrolimus, cyclosporine, mycophenolate, azathioprine, sirolimus, everolimus, and corticosteroids.

© 2020 D'Arcy ME et al. *JAMA Dermatology.*

eTable 2. Association Between Demographic or Clinical Factors and Cutaneous SCC Among Individuals With at Least 360 Days of Follow-up, With Medication Exposure Lagged 360 Days

Characteristic	aHR [a]	95% confidence interval	
		Lower	Upper
Sex			
Female (reference)	1.00	-	-
Male	1.97	1.69	2.31
Age, for each increasing age category [b]	1.54	1.43	1.66
Transplant Reason			
Chronic obstructive pulmonary disease (reference)	1.00	-	-
Idiopathic Pulmonary Fibrosis	1.36	1.15	1.61
All other [c]	1.26	1.02	1.55
Smoking history			
No (reference)	1.00	-	-
Yes	1.24	1.05	1.46
Daily average annual ambient ultraviolet radiation in mW/m^2			
< 26.00	1.00	-	-
26.00-31.99	0.94	0.78	1.14
32.00-46.89	0.96	0.80	1.16
47.00+	1.19	0.93	1.35
Transplant number			
First (reference)	1.00	-	-
Second or higher	2.11	1.55	2.86
Voriconazole [d]	1.05	1.04	1.06
Ever itraconazole			
No (reference)	1.00	-	-
Yes	1.00	0.80	1.24
Unknown	0.92	0.68	1.24
Ever posaconazole			
No (reference)	1.00	-	-
Yes	0.98	0.71	1.35
Unknown	1.30	0.89	1.89
Ever other antifungal [de]			
No (reference)	1.00	-	-
Yes	0.96	0.75	1.24
Unknown	1.01	0.71	1.42

Abbreviations: aHR, adjusted hazard ratio

a Cox regression models are adjusted for all variables in the table. The baseline hazard was stratified by calendar years (2007-2008; 2009-2010; 2011-1012; 2013-2014; 2015-2016).
b Age was categorized as an ordinal variable corresponding to the following age ranges: < 30, 30-39,40-49, 50-59, 60-69, > 69 years
c. All other indications include: cystic fibrosis, other obstructive lung diseases, inflammatory and fibrotic lung, airway diseases, pulmonary hypertension and pulmonary vascular diseases, and other or unspecified conditions
d Cumulative voriconazole exposure is assessed in 30-day increments; duration of use categories correspond to approximately <33rd, 33rd-66th, 66th-90th, >90th percentiles
e Other antifungal medications include fluconazole, amphotericin B, caspofungin acetate, micafungin sodium, anidulafungin, and isavuconazonium sulfate.

© 2020 D'Arcy ME et al. *JAMA Dermatology.*

eTable 3. Association Between Demographic or Clinical Factors and Cutaneous BCC Among Individuals With at Least 360 Days of Follow-up, With Medication Exposure Lagged 360 Days

Characteristic	aHR [a]	95% confidence interval	
		Lower	Upper
Sex			
Female (reference)	1.00	-	-
Male	1.65	1.26	2.15
Age, for each increasing age category [b]	1.24	1.09	1.41
Smoking history			
No (reference)	1.00	-	-
Yes	1.51	1.11	2.05
Daily average annual ambient ultraviolet radiation in mW/m^2			
5.20-25.98	1.00	-	-
26.00-31.99	0.82	0.59	1.12
32.00-46.89	0.68	0.48	0.95
47.00-77.73	1.05	0.77	1.44
Ever [c] voraconazole			
No (reference)	1.00	-	-
Yes	0.91	0.64	1.30
Unknown	0.98	0.60	1.63
Ever [c] itraconazole			
No (reference)	1.00	-	-
Yes	1.81	1.29	2.55
Unknown	1.25	0.77	2.03
Ever [c] posaconazole			
No (reference)	1.00	-	-
Yes	1.78	1.09	2.89
Unknown	1.59	0.83	3.03
Ever [c] other antifungal [d]			
No (reference)	1.00	-	-
Yes	0.74	0.46	1.19
Unknown	0.53	0.29	0.96

Abbreviations: aHR, adjusted hazard ratio

a Cox regression models are adjusted for all variables in the table. The baseline hazard was stratified by calendar years (2007-2008; 2009-2010; 2011-1012; 2013-2014; 2015-2016).
b Cumulative voriconazole exposure is assessed in 30-day increments.
c Yes ever exposure refers to a person that had at least one 30 day interval up until the current interval with an observed exposure to the antifungal. No (reference) exposure includes individuals who were not exposed at the evaluation interval and were unexposed >75% of all prior intervals. Unknown includes intervals with no known exposure intervals and intervals with < 75% of true unexposed intervals
d Other antifungals include fluconazole, amphotericin B, caspofungin acetate, micafungin sodium, anidulafungin, and isavuconazonium sulfate.

© 2020 D’Arcy ME et al. *JAMA Dermatology.*
